# Supplementary material for: Potential relationship of the gut microbiome with testosterone level in men: a systematic review
Source: PeerJ. 2025 Apr 15;13:e19289. doi: 10.7717/peerj.19289 (PMC12007503; doi:10.7717/peerj.19289)
Supplement: Supplemental Information 2 [file peerj-13-19289-s002.docx]

**The intended audience is microbiologists, endocrinologists, and gender medicine experts who are developing mechanisms for the relationship between the microbiome and testosterone production. In addition, the results of this study can be used as a reference for the possibility of developing therapies that involve the microbiome in reducing testosterone levels.**
